# Simulation design for improvement of undergraduate nursing students’ experience of evidence-based practice: A scoping-review protocol

**DOI:** 10.1371/journal.pone.0260238

**Published:** 2021-11-18

**Authors:** Chi Eun Song, Aeri Jang

**Affiliations:** Department of Nursing, Nambu University, Gwangju, South Korea; PLOS ONE, UNITED STATES

## Abstract

Simulation may be an effective educational strategy for undergraduate nursing students to experience evidence-based practice. The aim of this scoping review is to explore such simulations to discover the design characteristics that best achieve this goal. In this review, we will consider studies in which the focus was on evidence-based practice-related simulation programs for undergraduate students in academic, clinical, or virtual settings. We will also focus on the active learning strategies applied in such simulation programs. This scoping review will be conducted in accordance with the Joanna Briggs Institute methodology. Studies will be searched in Medical Literature Analysis and Retrieval System Online (MEDLINE; PubMed), the *Cumulative Index to Nursing and Allied Health Literature* (CINAHL), the Education Resources Information Center (ERIC), and the Excerpta Medica database (EMBASE). Sources of unpublished studies/gray literature will not be included in this scoping review. Data extraction will be undertaken by using a data-extraction tool developed by the reviewers, based on the National League for Nursing Jeffries Simulation Theory. Via a narrative summary and tabulated results, we will describe how the simulation programs were designed or implemented in an undergraduate curriculum.

## Introduction

Simulation is a useful strategy in nursing education and has received increasing attention. As patient safety is an important issue in the clinical setting, clinical practicums are mostly focused on observational learning. Simulation is considered an alternative to practicums to improve students’ clinical competence [1]. Simulation is also presented as an appropriate educational method for the replacement of clinical practicums, as the number of clinical sites is limited [2]. In addition, simulations can be presented as online clinical courses in situations in which it is difficult to present clinical practicums or simulation-based education in a face-to-face manner, e.g., during the coronavirus disease 2019 pandemic [3,4]. Systematic reviews have indicated that high-fidelity patient simulations may be effective in improving nursing students’ knowledge and performance [5], enhancing their self-confidence in performing nursing care, and reducing anxiety [6]. An umbrella review also revealed that simulations may be more effective in improving students’ self-efficacy than other teaching methods; furthermore, students’ satisfaction with simulation-based education is high [7].

Evidence-based practice (EBP) is an essential competency for nurses [8]. An undergraduate nursing curriculum needs to facilitate the systematic development of students’ EBP knowledge and skills, as well as their attitudes toward EBP [9,10]. However, several barriers have been reported, especially in clinical practicums. Students want to experience the implementation of EBP in a clinical setting, but are discouraged by nurses’ lack of EBP competencies or negative attitudes toward EBP [11]. Organization-related barriers, such as a lack of authority to change patient care or a lack of time and resources in the clinical setting, reportedly impede the improvement of students’ EBP knowledge and skills, as well as their attitudes toward EBP [12]. Simulation-based education is considered a useful way to reduce the gap between EBP theory and practice and overcome challenges in preparing students to implement EBP during their clinical practicum [13].

The design of the simulation affects student learning outcomes [14] and may influence students’ experience of EBP implementation in a simulated context. According to the National League for Nursing (NLN) Jeffries Simulation Theory, simulation design includes the learning objectives, elements of physical and conceptual fidelity, participant and observer roles, progression of activities, and briefing/debriefing strategies [15]. Owen et al. [16] designed a simulation for the practice of clinical decision-making with a focus on patient safety, by using upper-level nursing students as standardized patients. Foronda et al. [17] used a virtual simulation after a didactic class to enhance cognitive and affective knowledge about EBP in the care of patients with hypertension.

As simulation programs can be designed in various ways, a review of simulations designed to provide EBP experience to undergraduate students may provide basic data for the optimal design of EBP-related simulation programs. A preliminary search of Medical Literature Analysis and Retrieval System Online (MEDLINE; PubMed), *The Cochrane Database of Systematic Reviews*, and *JBI Evidence Synthesis* was conducted, and we identified no existing or ongoing systematic reviews or scoping reviews on this topic.

Our review questions are as follows:

- How are simulation programs designed to enable undergraduate nursing students to experience EBP?
- What active learning strategies are used in such programs to change evidence into practice?

## Materials and methods

This scoping review will be conducted in accordance with the Joanna Briggs Institute methodology [18]. The inclusion criteria are defined based on the population, concept, and context paradigm.

### Inclusion criteria

#### Participants

In the scoping review, we will consider studies in which the participants were nursing students in a baccalaureate program. Studies of simulation programs for undergraduate nursing students and clinical staff will be included. Studies of simulation programs for graduate (magisterial or doctoral) or practicing (licensed vocational nurses, registered nurses, advanced practice nurses, or the equivalent) nurses will be excluded. Studies of inter-professional simulations in which undergraduate nursing students participated for the cultivation of non-technical skills will also be excluded.

#### Concept

The concept of interest for this scoping review is the design of simulation programs for the experience of EBP by undergraduate nursing students. Studies in which the strategies for EBP implementation in a simulation program is described will be included in this review. Studies related to the development of simulation evaluation tools will be excluded.

#### Context

In this review, we will consider studies in which simulation programs were presented in academic or clinical settings, as well as studies of virtual simulations. Studies related to scenario development will be excluded, as well as those of simulation-based learning for simple knowledge transfer in research methodology or nursing theory courses, where not related to EBP.

#### Types of sources

In this scoping review, we will not limit studies based on design. Both experimental and quasi-experimental study designs will be considered. In addition, observational and descriptive studies will be considered for inclusion, as well as qualitative studies with content related to our review questions. However, all reviews will be excluded, as well as opinion or editorial articles in which the simulation design are not described.

### Methods

#### Search strategy

We will search for relevant published studies. An initial, limited search of MEDLINE and the *Cumulative Index to Nursing and Allied Health Literature* (CINAHL) was undertaken to identify articles on the topic. The text and the index terms in relevant articles were used to develop the full search strategy for MEDLINE (Table 1). The search strategy will be adapted for each included database. The reference lists of all included articles will be screened for additional studies. Studies published since 2000 in any language will be included. The databases to be searched include MEDLINE, CINAHL, the Education Resources Information Center (ERIC), and the Excerpta Medica database (EMBASE). Unpublished studies/gray literature will not be included in this scoping review.

**Table 1:** Search strategy for MEDLINE (PubMed).

#	query
1	(education, nursing, baccalaureate[MeSH Terms]) OR (students, nursing[MeSH Terms])
2	"nursing student*"[Title/Abstract] OR "undergraduate"[Title/Abstract] OR "Pre-registration nursing"[Title/Abstract] OR "Baccalaureate"[Title/Abstract] OR "Nursing"[Title/Abstract]
3	#1 OR #2
4	"Computer Simulation"[MeSH Terms] OR "Patient Simulation"[MeSH Terms] OR "High Fidelity Simulation Training"[MeSH Terms] OR "Simulation Training"[MeSH Terms] OR "Virtual Reality"[MeSH Terms]
5	"simulat*"[Title/Abstract] OR "Virtual"[Title/Abstract] OR "High-fidelity"[Title/Abstract] OR "Manikins"[Title/Abstract]
6	#4 OR #5
7	"Evidence-Based Practice"[MeSH Terms] OR "Evidence-Based Nursing"[MeSH Terms]
8	"evidence"[Title/Abstract] OR "Evidence-based"[Title/Abstract] OR "appraisal"[Title/Abstract] OR "application"[Title/Abstract] OR "EBP"[Title/Abstract] OR "inquiry"[Title/Abstract] OR "clinical question*"[Title/Abstract] OR "decision-making"[Title/Abstract] OR "guideline*"[Title/Abstract]] OR "Research"[Title/Abstract] OR "Management"[Title/Abstract]
9	#7 OR #8
10	#3 AND #6 AND #9; Filters: from Mar, 2000

#### Study/Source of evidence selection

Following the search, all identified citations will be collated and uploaded into EndNote X8 8.2 (Clarivate Analytics, Philadelphia, PA, USA), and duplicates will be removed. Titles and abstracts will be screened by one reviewer for assessment against the inclusion and exclusion criteria for the review. The full text of the selected citations will be assessed in detail against the study criteria by two independent reviewers. Articles written in languages other than Korean or English will be translated by a professional translator or Google Translate (Google LLC, Menlo Park, CA, USA). The reasons for exclusion of full-text articles will be reported in the scoping review. Any disagreements that arise between the reviewers in the selection process will be resolved through discussion. The results of the search and the study inclusion process will be reported in full in the final scoping review and presented in a Preferred Reporting Items for Systematic Reviews and Meta-Analyses extension for Scoping Reviews (PRISMA-ScR) flow diagram [19].

#### Data extraction

Data will be extracted by one reviewer (Dr. Song) using a data-extraction tool developed by the reviewers and will be verified by another reviewer (Dr. Jang). The extracted data will include specific details about the authors, year of publication, and country of origin. The main data to be extracted, based on the NLN Jeffries Simulation Theory [15], is as follows: context (academic, practice, or virtual), background (the goals of the simulation, how the simulation fits within the curriculum), design (the specific learning objectives, fidelity, and briefing/debriefing strategies), outcomes (related to participants, patients, and system areas), and key findings relevant to the review question, including the active learning strategy. A pilot review will be performed with the first 5 to 10 included studies in this scoping review.

The data-extraction tool can be modified and revised as necessary during the process of data extraction. The modifications will be described in the final scoping review. Any disagreements that arise between the reviewers in the data extraction process will be resolved through discussion.

#### Ethical consideration

This scoping review was exempted from approval by the Institutional Review Board of Nambu University, Gwangju, South Korea (1041478-2021-HR-029).

## Results

The data will be presented in tabular form according to core elements of the NLN Jeffries Simulation Theory. Data that can be quantitatively converted will be presented using descriptive statistics. A narrative summary and tabulated results will be used to describe the characteristics of the simulation design so that students can experience EBP and the active learning strategy is applied to problem-solving processes.

## Discussion

EBP competency is an essential skill for undergraduate nursing students to have acquired upon graduation [20]. However, because of a lack of human and material resources during their clinical practicums, not all students have the opportunity to properly apply the EBP for which they have learned the theory [11,13]. In addition, because of the coronavirus disease 2019 pandemic, face-to-face clinical practicums and simulation-based education has become more difficult to present [3]. This scoping review is intended to clarify ways in which simulations can be used to promote undergraduate nursing students’ implementation of EBP.

Based on data extracted according to the NLN Jeffries Simulation Theory, which has seen wide use in simulation-based education [21], we will discuss the optimal simulation design for students to experience EBP. In particular, we will identify and discuss the active learning strategies used in the design of the simulations to change evidence into practice, an important step in the EBP process.

Any changes to the content of the protocol made during the scoping review will be specified in detail, and the limitations of the review will be described.

## Conclusions

This scoping review will be performed to explore the use of simulations in the improvement of EBP competencies in undergraduate nursing students. It is expected to provide useful information on the design of simulation-based education and research to best achieve that goal.

## Supporting information

S1 Checklist PRISMA-P 2015 checklist. (DOCX)
